# Changes in protein structure monitored by use of gas‐phase hydrogen/deuterium exchange

**DOI:** 10.1002/pmic.201400440

**Published:** 2015-03-18

**Authors:** Helen S. Beeston, James R. Ault, Steven D. Pringle, Jeffery M. Brown, Alison E. Ashcroft

**Affiliations:** ^1^ Astbury Centre for Structural Molecular Biology & Faculty of Biological SciencesUniversity of LeedsLeedsUK; ^2^ Waters CorporationWilmslowUK

**Keywords:** Hydrogen/deuterium exchange‐MS, Ion mobility spectrometry, Protein folding, Secondary structure, Technology

## Abstract

The study of protein conformation by solution‐phase hydrogen/deuterium exchange (HDX) coupled to MS is well documented. This involves monitoring the exchange of backbone amide protons with deuterium and provides details concerning the protein's tertiary structure. However, undesired back‐exchange during post‐HDX analyses can be difficult to control. Here, gas‐phase HDX‐MS, during which labile hydrogens on amino acid side chains are exchanged in sub‐millisecond time scales, has been employed to probe changes within protein structures. Addition of the solvent 2,2,2‐trifluoroethanol to a protein in solution can affect the structure of the protein, resulting in an increase in secondary and/or tertiary structure which is detected using circular dichroism. Using a Synapt G2‐S ESI‐mass spectrometer modified to allow deuterated ammonia into the transfer ion guide (situated between the ion mobility cell and the TOF analyser), gas‐phase HDX‐MS is shown to reflect minor structural changes experienced by the proteins β‐lactoglobulin and ubiquitin, as observed by the reduction in the level of deuterium incorporation. Additionally, the use of gas‐phase HDX‐MS to distinguish between co‐populated proteins conformers within a solution is demonstrated with the disordered protein calmodulin; the gas‐phase HDX‐MS results correspond directly with complementary data obtained by use of ion mobility spectrometry‐MS.

**Abbreviations**
CD: circular dichroism
HDX: hydrogen/deuterium exchange
IMS: ion mobility spectrometry
TFE: 2,2,2‐trifluoroethanol

## 1 Introduction

As the number of protein‐related therapeutics under development increases, consideration must be given to their method of analysis, not only in terms of establishing the expected amino acid sequence and purity, but also with a view to monitoring variations in their 3D structure which are likely to alter functional behaviour. There are similar requirements for biological reference materials employed within the clinical community to ensure comparable results between laboratories 1. Hence, there is a continuing quest to establish analytical methods which are accurate, rapid, reproducible, require minimal quantities of sample and generate the maximum amount of information in a single experiment.

ESI‐MS is widely accepted as an invaluable biophysical technique for the study of proteins. ESI‐MS can not only measure a protein's molecular mass but also provide information on its conformational properties by means of the charge state distribution of ions produced 2, 3, 4. In general, the observation of low charge state ions indicates the presence of a structured protein in the gas phase, whilst higher charge state ions are associated with protein unfolding due to repulsive Coulombic interactions. In practice, a number of co‐populated protein conformers can be detected within an ESI charge state distribution, depending on both the solution phase of the protein prior to analysis (pH, ionic strength) and the instrumental parameters used to ionise and analyse the protein. The use of MS/MS to sequence proteins either in their intact state or following proteolytic digestion is a cornerstone of proteomics 5, 6 and, in the case of biomolecular complexes, both stoichiometry and stability can be ascertained using MS/MS 7, 8. Recent advances in coupling ESI‐MS to ion mobility spectrometry (IMS) commercially 9, 10 have permitted protein folding and assembly reactions to be monitored in real time routinely, with ESI‐IMS‐MS providing information about both mass and shape (via the rotationally averaged collision cross‐sectional area) of individual species within complex mixtures in a single, rapid experiment 11, 12, 13, 14, 15.

Interfacing ESI‐MS with solution‐based chemical modification methods can provide further information about the 3D structure of proteins 16, 17. In particular, solution‐phase hydrogen/deuterium exchange (HDX) followed by proteolysis and HPLC‐ESI‐MS(/MS) analysis of the peptide fragments has become established for probing the structure, dynamics and folding of proteins by monitoring the deuterium replacement of individual hydrogen atoms over time 18, 19, 20, 21. Solution‐phase HDX is a complex process, the kinetics of which depends on the dynamics of the protein and also on the intrinsic rate of hydrogen exchange. In solution, exposure of a protein to deuterated water induces a rapid amide H to D exchange in disordered regions that lack stable hydrogen bonding. Regions of the protein which are folded are more protected from HDX, resulting in slow exchange that is mediated by any structural dynamics of the protein 22. HDX is usually carried out at ∼pH 7 whereby the protein maintains its tertiary structure and the exchange rate is optimal. The exchange times for the most solvent‐exposed backbone amide hydrogens are >10 ms at pH 7. However, the labile hydrogen atoms situated on the amino acid side chains exchange more rapidly, in the order of <1–10 ms, but deuteration at these sites cannot be monitored in these experiments due to the rapid back‐exchange of D to H during post‐exchange analyses. Solution‐phase HDX, with the aid of post‐exchange proteolysis followed by MS/MS to measure the HDX of individual amino acid residues, provides details about conformational changes and the formation of new interfaces during folding events 20, 21, 23, 24. The caveat of solution‐phase HDX is primarily the need to minimise unwanted D to H back‐exchange at the amide backbone sites, which is achieved to some extent by rapidly lowering both the temperature and pH of the solution to quench the HDX reaction followed by immediate HPLC‐ESI‐MS(/MS) analysis.

As an alternative approach for assessing structural changes to a protein, gas‐phase HDX is gaining momentum. Since the early work on protonated benzene derivatives 25, gas‐phase HDX has been employed to study glycine oligomers 26, peptides 27 and proteins 28, 29, inter alia. To date, studies which have reported the detection of protein conformational changes include a comparison of an *Escherichia coli* gene product, UmuD, a 139 amino acid residue protein, with its cleavage product UmuD’, in which gas‐phase HDX showed a greater uptake of D for the truncated protein 30. Recent studies elsewhere following in‐house modifications to a commercially available ESI‐MS Q‐TOF instrument equipped with IMS reported benefits of gas‐phase HDX including controlled labelling times (from 0.1 to 10 ms) and avoidance of the back‐exchange issues encountered when quenching post‐exchange and during the subsequent MS analyses when employing solution‐phase HDX 31, 32. These studies reported the deuterium exchange of the fast‐exchanging amino acid side chain hydrogen atoms, rather than the amide backbone hydrogens, on account of the short exchange times employed and demonstrated gas‐phase deuterium uptake for a number of peptides plus the protein ubiquitin, together with a comparison of wild‐type lysozyme with its disulphide bridge‐reduced analogue 31.

Here, we have used a similar, in‐house‐modified Q‐TOF mass spectrometer to perform gas‐phase HDX studies to investigate specifically the feasibility of detecting solution‐based conformational changes of a protein, starting from the folded protein in solution. We have used addition of the solvent 2,2,2‐trifluoroethanol (TFE) to the protein dissolved in aqueous ammonium acetate to induce conformational changes. The effects of co‐solvents including alcohols such as methanol, TFE and 1,1,1,2,2,2‐hexafluoro‐2‐propanol on protein conformation have been reported 33. In particular, TFE has been used at low concentrations to study the conformational structure of proteins due to its ability to stabilise secondary structure, particularly α‐helices by strengthening hydrogen bonds, and its effect on the folding pathways of proteins 34, 35. However, at higher concentrations such solvents are known to partially or fully unfold a protein 33. In our studies, any conformational changes induced by addition of TFE to the protein in solution were monitored using both solution‐phase circular dichroism (CD) spectroscopy and gas‐phase HDX coupled to ESI‐MS. A correlation between the two techniques was found in that distinct structural changes could be observed in both. In these examples, the structural change of the protein would not have been detected from the ESI‐MS charge state distribution alone, but was clearly observed using gas‐phase ESI‐HDX‐MS. We have also shown that gas‐phase HDX‐MS is successful in resolving co‐populated protein conformers, and for the first time have shown that the number of conformers detected by gas‐phase HDX is consistent with the conformeric signature detected by ESI‐IMS‐MS.

## 2 Materials and methods

### 2.1 Proteins

[Glu^1^]‐Fibrinopeptide B, bovine ubiquitin, bovine β‐lactogl‐obulin and bovine calmodulin (Sigma‐Aldrich, Gillingham, Dorset, UK) were each dissolved at a concentration of 0.2 mg/mL in 50 mM ammonium acetate buffer. Fifteen percent TFE (Sigma‐Aldrich) was added as stated.

### 2.2 CD analysis

CD analyses were performed using a Chriscan spectropolarimeter (Applied PhotoPhysics Ltd., Leatherhead, Surrey, UK). Each experiment was repeated three times and the results averaged to give the final result. A background scan containing buffer only was subtracted from the protein data.

### 2.3 MS analyses

In‐house‐pulled gold‐palladium coated capillaries 36 were used to infuse 5 μM protein in 50 mM ammonium acetate solution into the nano‐ESI source of a quadrupole‐IMS‐TOF Synapt G2‐S mass spectrometer (Waters Corpn., Manchester, UK) using a capillary voltage of 1.8 kV. The source temperature was set at 80°C, the cone voltage at 20 V, the transfer wave velocity at 247 m/s and the transfer wave height at 0.2 V. Caesium iodide cluster ions were used to calibrate the *m/z* scale. Measured masses of the proteins were within 0.01% of the theoretical mass. All data were processed using the MassLynx v4.1 software supplied with the instrument.

Gas‐phase HDX was carried out following modification of the Synapt G2‐S to allow the admission of deuterated ammonia (ND_3_; Spectra Gases Ltd., Cambridge, Cambridgeshire, UK) into the transfer ion guide situated between the IMS device and the TOF analyser, as reported elsewhere 31, 32 and shown in Supporting Information Fig. 1.

For HDX analyses, ND_3_ gas was infused into the transfer ion guide (pressure 5 × 10^−3^ mbar measured by the Pirani gauge) of the mass spectrometer. To maintain consistency between gas‐phase HDX‐MS experiments, glu‐fibrinopeptide B was analysed first as described elsewhere 31 and the ND_3_ gas pressure adjusted until the level of deuterium uptake was optimised (i.e. ten deuterium atoms exchanged for the 2+ charge state ions at *m/z* 785.9) under the experimental conditions described here. For assessing the level of deuteration incorporated into the proteins under study, the *m/z* peaks were smoothed (Savitszky–Golay algorithm) and centroided using MassLynx v4.1 software.

For ESI‐IMS‐MS analysis of calmodulin, a transfer collision energy of 2 V, IMS helium gas flow of 2 mL/min and nitrogen gas flow of 35 mL/min were used. Calibration of the measured IMS drift times was performed as described previously 37.

## 3 Results

### 3.1 Monitoring minor conformational changes effected by solvent

**Figure 1:**
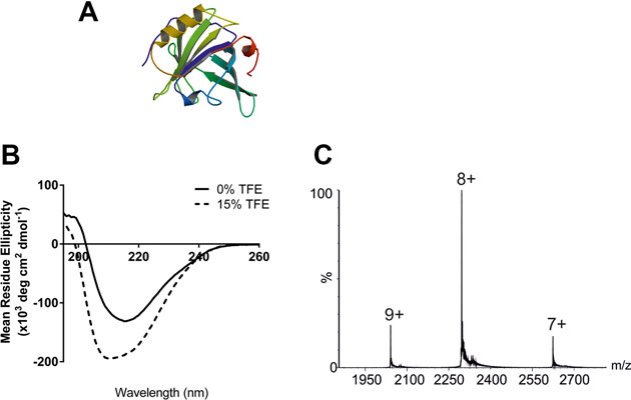
(A) Ribbon diagram of bovine β‐lactoglobulin (PDB: 3BLG 38); (B) β‐lactoglobulin CD spectra in 50 mM ammonium acetate in the absence of TFE (black line) and with 15% TFE added (dashed line); (C) ESI‐MS spectrum of bovine β‐lactoglobulin in 50 mM aqueous ammonium acetate.

Bovine β‐lactoglobulin, a 162‐residue, 18 281.3 Da globular protein, is the major whey protein in cow's milk and has a structure dominated by nine β‐strands together with one major, plus four short, α‐helices 38, 39 (Fig. 1A). NMR experiments carried out elsewhere on β‐lactoglobulin in the presence of TFE indicated that when exposed to this solvent, the protein exhibited significant α‐helical structure and this non‐native state was termed the ‘TFE‐state’ 39, 40. A co‐operative transition from the native to α‐helical structure has been reported using ∼15% TFE at pH 2 41. Using ESI‐MS, the formation of an intermediate with non‐native helical structure during β‐lactoglobulin unfolding effected by the addition of 16% TFE to an aqueous solution (pH 2) has also been observed by means of its extended charge state distribution of ions 42.

Here, we have investigated whether the TFE solution‐induced β‐lactoglobulin structure can be probed further by use of gas‐phase HDX‐MS. Firstly, the effect of solvent composition on the solution structure of β‐lactoglobulin was evaluated. Following addition of 15% TFE to the protein in 50 mM aqueous ammonium acetate at neutral pH, the protein structure was monitored by CD spectroscopy. Changes in the CD spectra indicative of increased α‐helicity can clearly be observed (Fig. 1B).

ESI‐MS analysis of β‐lactoglobulin in 50 mM aqueous ammonium acetate alone generated a narrow charge state distribution of ions ranging from 7+ to 9+ indicating a compact structure (Fig. 1C). With the addition of 15% TFE to the solution, very low abundance (<5%) 10+ and 11+ ions were detected also (data not shown). However, the predominant charge state ions observed for β‐lactoglobulin were 7+, 8+ and 9+ regardless of whether the protein was dissolved in ammonium acetate solution in the absence or presence of TFE, and from these ESI‐MS data alone it was not possible to observe any significant structural changes due to the addition of TFE.

Higher levels (i.e. >15%) of TFE were found to change the ESI‐MS charge state distribution of β‐lactoglobulin significantly, hence 15% TFE was used to increase the level of solution‐phase α‐helical structure in β‐lactoglobulin without changing the three dominant charge states in the mass spectrum. This permitted a direct comparison of the same charge state ions under different solution conditions by gas‐phase HDX‐MS.

**Figure 2:**
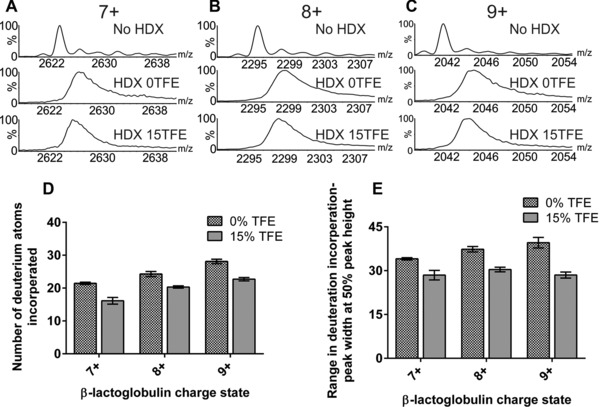
ESI‐MS gas‐phase HDX of β‐lactoglobulin. ESI‐MS spectra showing the degree of deuterium incorporation experienced by β‐lactoglobulin in the absence of TFE (0TFE) and with 15% TFE (15TFE) added for (A) the 7+ ions; (B) the 8+ ions and (C) the 9+ ions. (D) The mean deuterium incorporation of the 7+, 8+ and 9+ ions in the absence of TFE and with 15% TFE added; (E) the range of deuterium incorporation into the 7+, 8+ and 9+ ions estimated by identifying the peak width at 50% peak height. All bar charts represent the mean values of three replicates; error bars are the standard deviation of the mean.

To determine whether gas‐phase HDX‐MS can reveal subtle changes in solution‐phase protein structure, β‐lactoglobulin was analysed from ammonium acetate solution, both in the absence of TFE and with 15% TFE added. The level of HDX occurring for the 7+, 8+ and 9+ charge state ions of β‐lactoglobulin is illustrated by the shifting of the *m/z* peaks to higher values when exposed to ND_3_ gas in the transfer ion guide (Fig. 2A–C, respectively). The number of deuterium atoms incorporated into each charge state has been estimated (Fig. 2D). There is a clear correlation between increased levels of HDX with increasing charge state, with 21 hydrogens having undergone exchange in the case of the 7+ ions, 24 hydrogens exchanging for the 8+ ions and 28 hydrogens undergoing exchange for the 9+ ions of β‐lactoglobulin. There are 118 labile hydrogen atoms on the amino acid side chains of the neutral protein and so, under the conditions employed here, ∼17% are exchanged for deuteriums in the case of the 7+ ions (i.e. a total of 118 plus nH^+^, where n is the charge on the protein 28). The addition of 15% TFE to the protein solution led to a small but reproducible reduction in the level of HDX, with four or five fewer hydrogens undergoing exchange for all three charge states, consistent with a change in the protein's structure which gives rise to lower ND_3_ accessibility of some amino acid side chains.

To gauge whether the addition of TFE to the protein solution affects protein dynamics, the spread of deuterium atoms incorporated for each charge state was assessed by taking the peak width of the isotopic distribution of each charge state at 50% of the peak height and estimating the distribution of deuterium atoms incorporated into the protein (Fig. 2E). For example, for the 9+ ions (*m/z* 2038–2054), the width of the charge state peak at 50% of the peak height decreased by ∼20% from a range of 38 deuterium atoms in the absence of TFE to 30 deuteriums in the presence of 15% TFE (Fig. 2C and E). Similarly, for the 7+ and 8+ charge state ions, upon addition of 15% TFE to β‐lactoglobulin in aqueous ammonium acetate solution, the spread of deuterium incorporation was narrowed compared to the spread observed in the absence of TFE (Fig. 2E). This is consistent with the notion that TFE acts to stabilise the protein structure, making it less dynamic and thus reducing the range of labile hydrogens exposed to ND_3_ gas.

**Figure 3:**
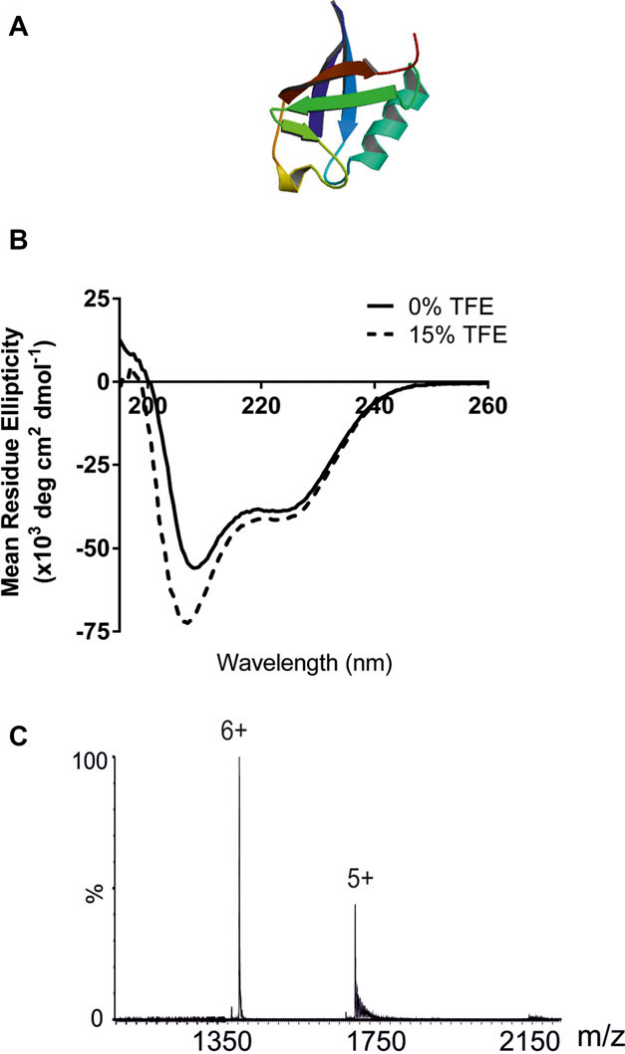
(A) Ribbon diagram of bovine ubiquitin (PDB: 1UBQ) 43; (B) circular dichroism spectra of ubiquitin in 50 mM ammonium acetate solution in the absence of TFE (black line) and with 15% TFE added (dashed line); (C) ESI‐MS spectrum of ubiquitin in 50 mM aqueous ammonium acetate solution.

Ubiquitin is a 76 amino acid residue, 8564.9 Da, regulatory protein found in most tissues of eukaryotic organisms. Its role is to ubiquitinate other proteins, a modification which tags the substrate protein for degradation. The tertiary structure of bovine ubiquitin comprises both α‐helices and β‐sheets (Fig. 3A) 43 and ubiquitin has 144 labile hydrogens, split almost equally between the protein backbone and the amino acid side chains, the latter which are potential exchangeable sites for these experiments 44. Gas‐phase HDX of ubiquitin using deuterated methanol has been reported using an ion trap mass spectrometer 28 and also with ND_3_ on a Q‐TOF instrument 31. In both cases, the behaviour of highly charged ions was scrutinised. The former approach focussed on the 10+ ions of which approximately 26% of the potential exchangeable sites underwent HDX within 1 s, whilst for the 7+ ions this percentage dropped to 19% 28. In the latter report, ubiquitin was analysed at low pH (50% aqueous ACN with 0.1% formic acid) and 50% of all labile side chain hydrogen atoms, or approximately 25% of all labile hydrogens, were observed to exchange within 0.33 ms 31.

To determine how the presence of TFE can influence the solution‐phase structure of ubiquitin, firstly CD spectroscopy was performed on bovine ubiquitin dissolved in 50 mM ammonium acetate solution in the absence of TFE and in the presence of 15% TFE. A change in the secondary structure of ubiquitin was observed as illustrated by the differences in the CD spectra which indicate a distinct increase in the α‐helical content of the protein (Fig. 3B).

ESI‐MS analysis of ubiquitin in the absence of TFE generated a charge state distribution which included primarily the 5+ and 6+ charge state ions and this distribution did not change upon addition of 15% TFE (Fig. 3C). Previously, ESI‐IMS‐MS data have indicated that the 4+–6+ ions inclusive have a collision cross‐section consistent with the protein's crystal structure and hence retain a compact structure 45, whilst the 7+ ions and the 11+ ions have been shown (independently) to be expanded in the gas phase using ECD‐FTICR 46, and ESI‐IMS‐MS 31, respectively. Thus, under our conditions, a compact protein conformation is observed.

**Figure 4:**
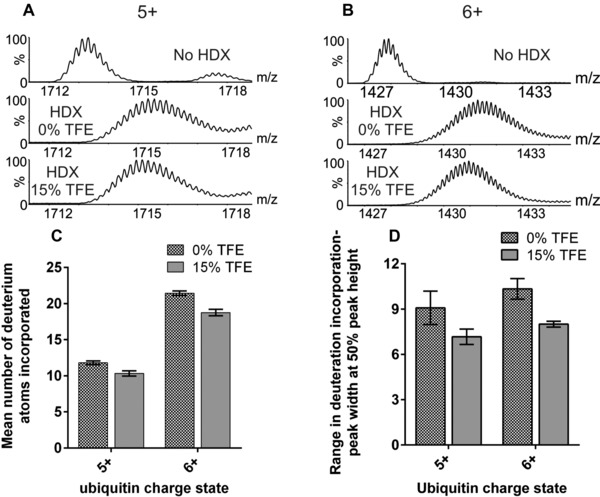
ESI‐MS gas‐phase HDX of bovine ubiquitin. ESI‐MS spectra showing the degree of deuterium incorporation experienced by ubiquitin in the absence of TFE and with 15% TFE added for (A) the 5+ ions and (B) the 6+ ions. (C) The mean incorporation of deuterium atoms in the 5+ and 6+ ions of ubiquitin in the absence of TFE and with 15% TFE added; (D) the range of deuterium incorporation into the 5+ and 6+ ions estimated by identifying the peak width at 50% peak height. All bar charts are the mean values of three replicates; error bars are the standard deviation of the mean.

Next, ubiquitin was subjected to gas‐phase ESI‐HDX‐MS to investigate whether inducing structure in ubiquitin in solution by the addition of 15% TFE can be detected. Both the 5+ and 6+ charge state ions showed a slightly lower degree of HDX when 15% TFE was added to the protein in 50 mM aqueous ammonium acetate solution (Fig. 4A and B). The number of deuterium atoms incorporated into ubiquitin is illustrated graphically (Fig. 4C). For the 5+ ions, 12 deuterium atoms out of a possible 65 (i.e. ∼17%) were incorporated into the protein in the absence of TFE, whilst on addition of 15% TFE to the solution, this number was reduced to ten (i.e. ∼14%). A similar small but measurable reduction in deuterium uptake was observed for the 6+ ions: 21 deuterium atoms (32%) were incorporated in the absence of TFE compared with 19 deuterium atoms (29%) upon addition of 15% TFE (Fig. 4C). To assess the dynamics of ubiquitin, the peak widths at 50% peak height of the isotopic distributions of the 5+ (*m/z* 1713–1717) and 6+ (*m/z* 1428–1433) ions were estimated. The addition of 15% TFE to the protein solution led to a reduced range of deuterium atoms being incorporated (Fig. 4D): the span of the deuterium incorporation of the 5+ ions decreased from a range of nine deuterium atoms to seven, and for the 6+ ions the decrease was from ten to eight deuterium atoms. Thus, the addition of TFE to ubiquitin in solution leads to a subtle change in protein structure which is reflected by a reduction in the number of hydrogens exchanged during gas‐phase HDX, in addition to a reduction in the spread of deuterium atoms incorporated.

Together, these observations indicate that gas‐phase HDX‐MS can be an effective method for recognising subtle solution‐phase protein structural changes which may otherwise be unnoticed from a simple ESI‐MS analysis.

### 3.2 Distinguishing co‐populated conformational families

**Figure 5:**
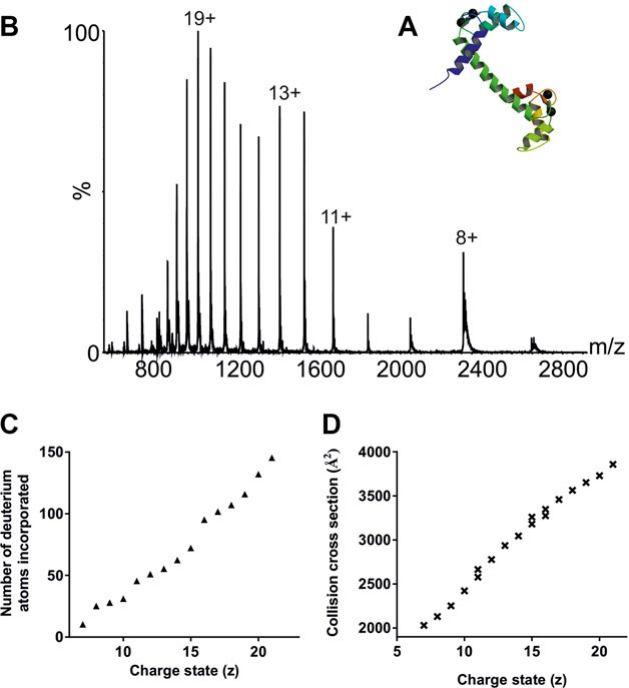
(A) Ribbon diagram of bovine calmodulin (PDB: 1OSA 50); (B) ESI‐MS spectrum of bovine calmodulin from 50 mM aqueous ammonium acetate solution; (C) number of deuterium atoms incorporated from gas‐phase HDX as a function of charge state; (D) ESI‐IMS‐MS estimated collision cross‐sections for calmodulin as a function of charge state.

Bovine calmodulin, a 168 amino acid residue protein of 18 870.0 Da, is the primary regulator of intracellular Ca^2+^ signalling in vivo. Calmodulin can bind specifically to more than 100 protein targets and thus requires a degree of structural flexibility to accomplish its functioning role 47 (Fig. 5A). The NMR solution structure of calmodulin indicates considerable backbone plasticity which is key to the protein's ability to bind a wide range of targets 48.

The conformational dynamics of calmodulin can be observed in its ESI‐MS mass spectrum which shows a wide range of charge states from 7+ to 22+ (Fig. 5B) when the protein is analysed from 50 mM aqueous ammonium acetate at neutral pH. Rather than a single Gaussian‐like profile, the charge state distribution of calmodulin indicates at least three co‐populated protein conformers centred on charge states 8+, 12+/13+ and 19+. In general, the higher charge states associated with protein ESI‐MS mass spectra signify more expanded, unfolded conformations and these structures are likely to undergo higher levels of HDX than the conformers associated with the lower charge states which are indicative of a more compact, folded protein structure (e.g. 31).

To investigate whether the co‐populated protein conformers observed can be distinguished using gas‐phase HDX‐MS, the incorporation of deuterium into calmodulin was measured as a function of charge state. By comparing each charge state with the number of deuterium atoms incorporated by those ions, it was found that in addition to the incremental increase in deuterium incorporation expected with the increasing number of charges on the protein ions, there are also more significant increases in deuteration levels between certain charge states, namely between the 10+/11+ and 15+/16+ pairs of ions, than may be expected if Coulombic repulsion was the only criterion to be considered (Fig. 5C). Three conformational families can be identified from the gas‐phase HDX results: one includes charge state ions 7+–10+, a second the 11+–15+ ions and a third the 16+–21+ ions. Calmodulin has 130 labile hydrogen atoms on its amino acid side chains which, depending on the conformation of the protein, could be amenable to HDX. The HDX levels for the three conformers detected are ten deuteriums, or ∼8% incorporation, for the most compact conformer based on the 7+ ions, 45 deuteriums (32% incorporation) based on the 11+ ions for the intermediate conformer and 96 deuteriums (66% incorporation) for the most extended conformer based on the 16+ ions. When assessing the range of deuterium incorporation of individual charge state ions, as opposed to the absolute amount of deuterium incorporation, certain charge state ions were observed to have a greater peak width (at 50% of the peak height) than others (Supporting Information Fig. 2 and Supporting Information Table 1). For example, the peak widths of the 10+ and 12+ ions are 2.73 and 2.75 on the *m/z* scale, respectively, compared with a peak width of 3.27 for the 11+ ions. One possible inference from these differences is that the 11+ ions have contributions from two conformers, the most compact and the partially expanded conformers described above, which leads to a larger range of deuterium incorporation. A similar observation can be made with the 14+ and 15+ ions, which have peak widths of 2.60 and 3.81 (*m/z*), respectively. Again, this could suggest that the 15+ ions have contributions from more than one protein conformer. All ions >15+ have peak widths ≥3.56, consistent with the increased dynamics of these more highly charged protein ions.

In parallel to the gas‐phase HDX experiments, ESI‐IMS‐MS was used as an alternative method to probe the conformational signature of calmodulin under the same solution conditions. From these data, the rotationally averaged collision cross‐section for each charge state from 7+ to 21+ was estimated 37, 49 (Fig. 5D). This method also identified three distinct, co‐populated conformational families of calmodulin under these conditions: a compact structure (7+–11+ ions; collision cross‐section 2040 Å^2^), a more extended structure (11+–16+ ions; collision cross‐section 2650 Å^2^) and a significantly larger structure (15+–21+; collision cross‐section 3250 Å^2^), which match very closely the conformational profile observed by gas‐phase HDX incorporation (Fig. 5C). Thus, gas‐phase HDX‐MS can be used to distinguish and map co‐populated protein conformers.

## 4 Discussion

As reported previously, gas‐phase HDX can be carried out successfully on a simply modified Q‐TOF mass spectrometer 31, 32. Here, we have shown that this method generates valuable information concerning the structural characteristics of proteins. The addition of 15% TFE to both β‐lactoglobulin and ubiquitin in aqueous ammonium acetate solution led to changes in each protein's solution‐phase structure, which were maintained in the gas phase and are reflected in a decrease in the number of hydrogen atoms exchanged following gas‐phase HDX‐MS. Further, the reduction in the spread of deuterium atoms incorporated suggests a decrease in the heterogeneity of the rapidly interconverting conformeric forms of the protein. For both β‐lactoglobulin and ubiquitin, the addition of 15% TFE did not change the ESI‐MS charge state distribution to any measureable extent and hence the subtle changes to the proteins’ secondary structures, which were clearly observed in solution by CD analyses, would have gone unnoticed if ESI‐MS alone had been used, without gas‐phase HDX.

The data presented show, for the first time, a direct correlation between ESI‐IMS‐MS and gas‐phase ESI‐HDX‐MS for the study of co‐populated protein conformers. Unlike β‐lactoglobulin and ubiquitin, calmodulin exhibits a wide charge state distribution of ions when analysed by ESI‐MS from aqueous ammonium acetate solution, indicating the co‐population of multiple conformers. These conformeric families can be separated using ESI‐IMS‐MS on account of the rotationally averaged collision cross‐section (i.e. shape) of their ions. Here, gas‐phase ESI‐HDX‐MS has been shown to deuterate co‐populated protein conformers to varying extents, depending on how compact or extended the individual species are. The number of calmodulin conformers present, and the charge state ions associated with each, is apparent from the HDX‐MS data. The conformational families distinguished using gas‐phase HDX are fully consistent with those observed by ESI‐IMS‐MS.

Gas‐phase HDX coupled with ESI‐MS offers a fast and reproducible method for highlighting changes in a protein's secondary structure that may not be apparent from the charge state distribution alone, and also for identifying co‐populated protein conformers. This technique, which uses picomolar amounts of protein and takes <2 min to perform each analysis, offers a potential method for verifying the conformeric heterogeneity of a protein and for monitoring conformational profile changes arising from disordered proteins during folding and functional events. Both applications have significant potential for biopharmaceutical purposes.

*The authors have declared no conflict of interest*.

## Supporting information

As a service to our authors and readers, this journal provides supporting information supplied by the authors. Such materials are peer reviewed and may be re‐organized for online delivery, but are not copy‐edited or typeset. Technical support issues arising from supporting information (other than missing files) should be addressed to the authors.

Figure S1
Figure S2
Table S1
