# Supplementary material for: Changes in protein structure monitored by use of gas‐phase hydrogen/deuterium exchange
Source: Proteomics. 2015 Mar 18;15(16):2842–50. doi: 10.1002/pmic.201400440 (PMC4973844; doi:10.1002/pmic.201400440)
Supplement: Supplementary file 1 — Figure S1 Figure S2 Table S1 [file PMIC-15-2842-s001.docx]

**Changes in protein structure monitored by use of gas-phase hydrogen/deuterium exchange**

Helen S. Beeston^1^, James R. Ault^1^, Steven D. Pringle^2^, Jeffery M. Brown^2^, Alison E. Ashcroft^1^*.

1. Astbury Centre for Structural Molecular Biology & Faculty of Biological Sciences, University of Leeds, Leeds, LS2 9JT, UK

2. Waters Corporation, Stamford Avenue, Altrincham Road, Wilmslow, SK9 4AX, UK

**Supplementary Information**

**Supplementary Figure 1.** Modifications to the Synapt G2-S Q-IMS-Tof mass spectrometer to allow the admission of deuterated ammonia into the transfer ion guide situated between the ion mobility separation device and the Tof m/z analyser; V = valve ^1,2^.


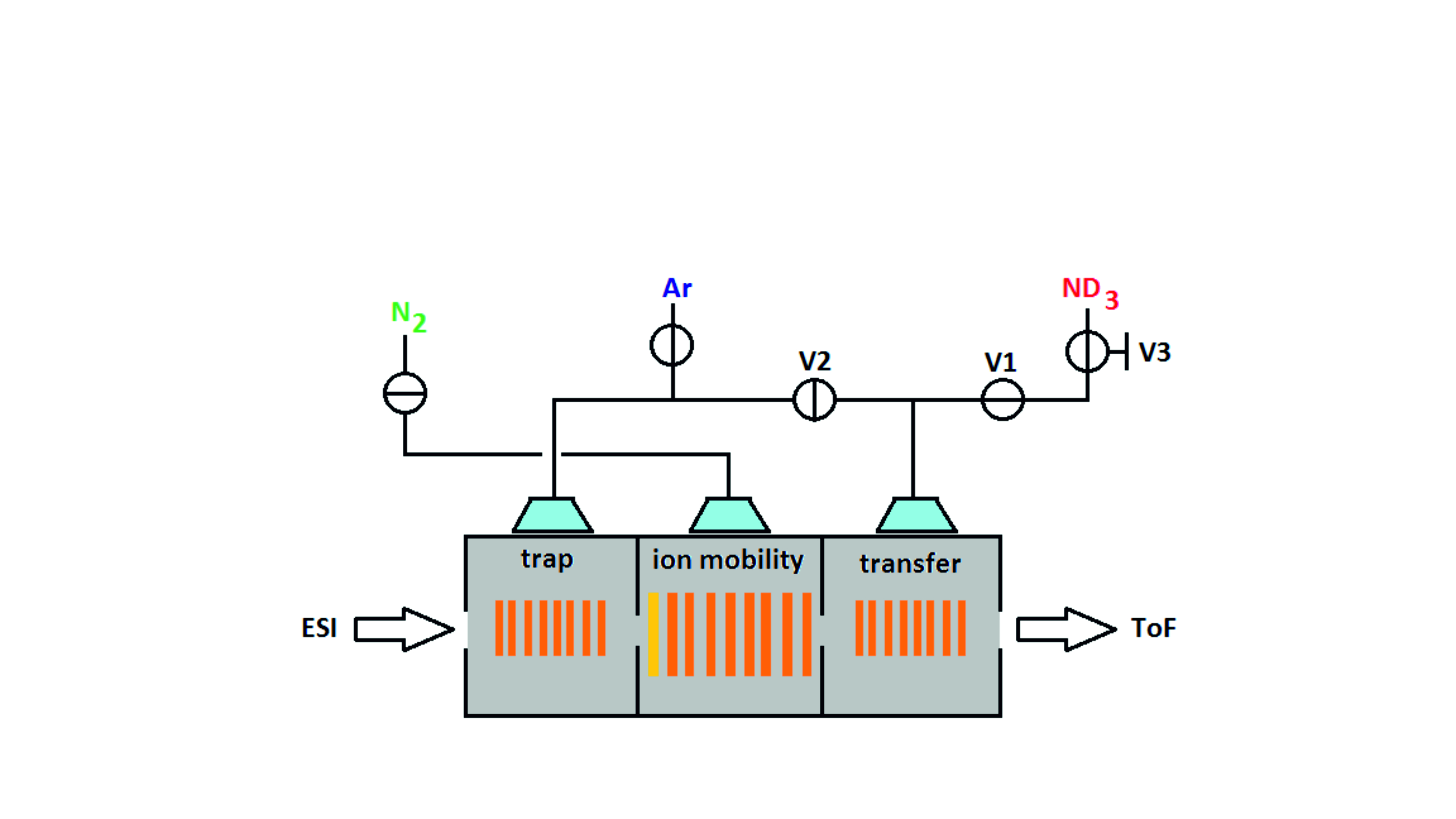


**Supplementary Figure 2.** (a) ESI-MS spectrum of bovine calmodulin from 50 mM aqueous ammonium acetate solution following gas-phase HDX; (b) 10+, 11+, 14+ and 15+ charge states ions of bovine calmodulin following gas-phase HDX showing *m/z*, charge state, and peak width at 50% height.

(a)

**
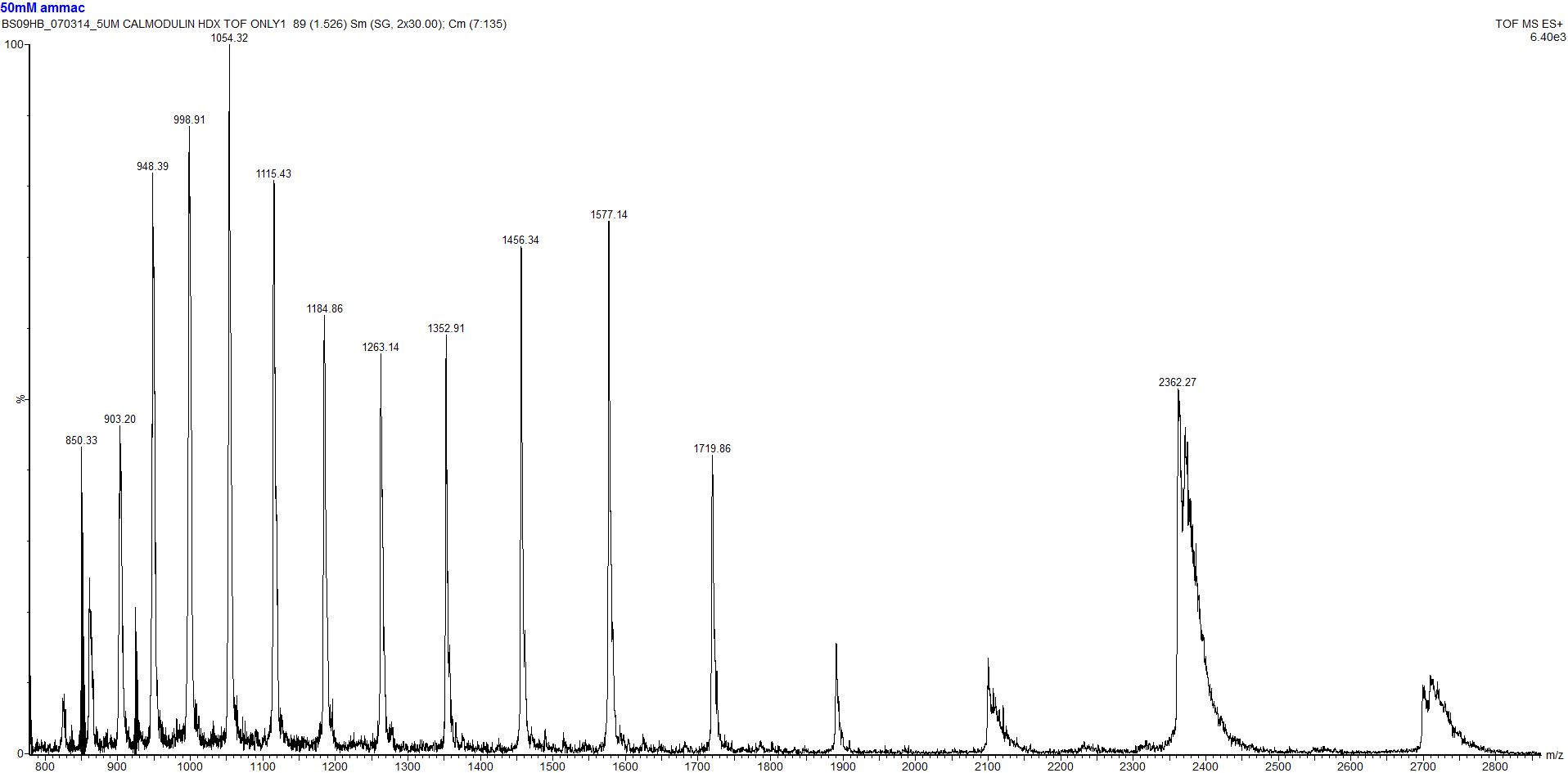
**

(b)

**
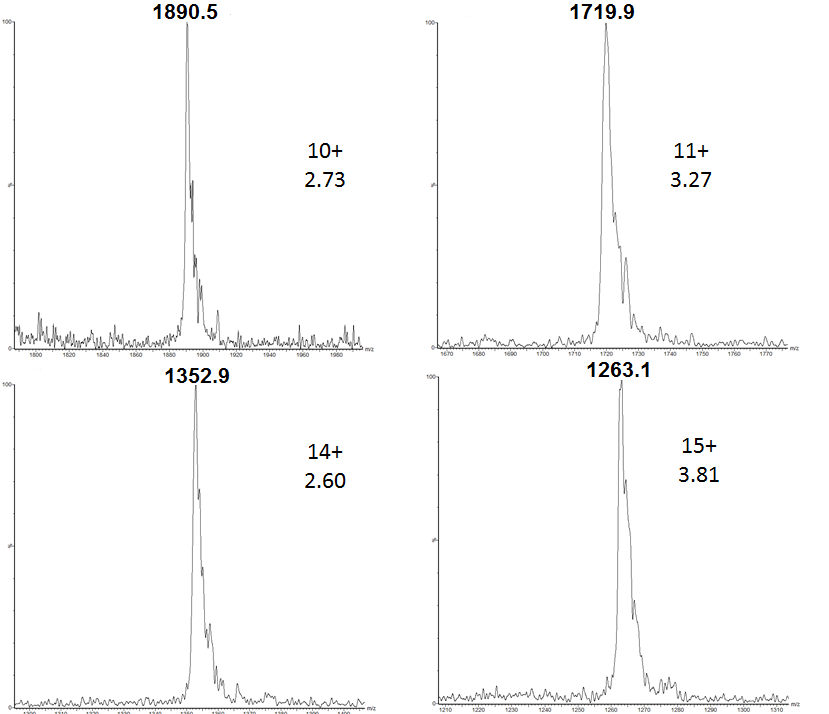
**

**Supplementary Table 1.** The peak width measured at 50% height for the bovine calmodulin multiply charged ions detected following gas-phase HDX.

| **Charge state ions** | **Peak width at 50% height**  **(*m/z*)** |
| --- | --- |
| **10+** | **2.73** |
| **11+** | **3.27** |
| **12+** | **2.75** |
| **13+** | **2.54** |
| **14+** | **2.60** |
| **15+** | **3.81** |
| **16+** | **3.60** |
| **17+** | **3.74** |
| **18+** | **3.56** |
| **19+** | **4.58** |

**References**

1. Rand, K. D., Pringle, S. D., Morris, M., Brown, J. M., Site-specific analysis of gas-phase hydrogen/deuterium exchange of peptides and proteins by electron transfer dissociation. *Anal. Chem.* 2012, *84*, 1931-1940.

2. Rand, K. D., Pringle, S. D., Murphy, J. P., Fadgen, K. E., Brown, J.*, et al.*, Gas-phase hydrogen/deuterium exchange in a traveling wave ion guide for the examination of protein conformations. *Anal. Chem.* 2009, *81*, 10019-10028.
